# The impact of co-fed plastic diet on *Tenebrio molitor* gut bacterial community structure

**DOI:** 10.1038/s41598-025-04805-8

**Published:** 2025-07-01

**Authors:** Larisa Ilijin, Dušanka Popović, Milica Živković, Dajana Todorović, Marija Mrdaković, Milena Vlahović, Vesna Perić-Mataruga

**Affiliations:** 1 Department of Insect Physiology and Biochemistry, Institute for Biological Research Siniša Stanković, National Institute of Republic of Serbia, University of Belgrade, Despot Stefan Blv. 142, 11060 Belgrade, Serbia; 2 Department of Ecology, Institute for Biological Research Siniša Stanković, National Institute of Republic of Serbia, University of Belgrade, Despot Stefan Blv. 142, 11060 Belgrade, Serbia; 3 Institute of Molecular Genetics and Genetics Engineering, University of Belgrade, Vojvode Stepe 444a, P.O. Box 23, 11010 Belgrade, Serbia

**Keywords:** Pollution remediation, Biotechnology, Microbiology

## Abstract

This study aimed to analyze the long-term impact of co-fed plastic diet on the bacterial community of self-sustaining laboratory populations of *T. molitor* fed with wheat bran with added polystyrene (PS), and low density polyethylene (LDPE) over a three year period. The most abundant phyla for all three populations were Firmicutes, Bacteroidota and Proteobacteria. PS group microbiota is similar to C group, pointing to a common bacterial species capable for degrading lignocellulose and PS, while consumption of LDPE caused a significant decrease of Bacteroidota and Actinobacteriota compared to both C and PS group, and Campylobacterota compared to PS group. A predictive metabolomics analysis recognized dTDP-L-rhamnose biosynthesis I in PS group as one of five unique pathways, while other five distinctive pathways, like peptidoglycan maturation, were linked to LDPE group. Further studies are needed to determine the plastic degrading properties of the detected bacteria. The results highlight *T. molitor’s* versatility in biotechnological applications.

## Introduction

Plastic pollution, characterized by a long time of decomposition, is one of the biggest environmental problems today. Due to the rapid growth of the human population, the amount of municipal plastic waste is high and persistent. In the last 15 years the production of plastics has shifted from the production of long-lasting plastics to single-use plastics (SUPs)^1^, with plastic packaging materials like polystyrene (PS) and low density polyethylene (LDPE) dominating^2^. PS is a styrene polymer most commonly used in the food industry for one-time, low-cost protective packaging. The widespread use of non-biodegradable PS has led to an increasing accumulation of PS waste in our environment, which is now one of the largest polluters of soils, lakes, rivers and oceans and marine organisms^3,4^. LDPE is a thermoplastic plastic made from the monomer ethylene. When exposed to constant sunlight, LDPE produces significant amounts of two greenhouse gases: methane and ethylene, contributing to carbon emission. It is widely used for manufacturing various food containers, plastic bags, kitchen wrap and is highly resistant to degradation^5^.

Since conventional methods for recycling plastic waste represent an additional danger to the ecosystem, biodegradation is increasingly recognized as a good solution which could contribute to plastic waste mitigation^6^. Not only organisms from the soil and water have the capacity to decompose plastic, it has been shown that plastic degrading bacteria exist even in the human gut^7,8^. Today, numerous representatives of actinomycetes, algae, fungi and bacteria, as well as many insects, are known for their ability to degrade plastic^9^. Given that, the yellow mealworm *T. molitor* (Coleoptera: Tenebrionidae), is not only a good source of protein for food and feed, but it is recognized for its potential to degrade PS^9–14^ and LDPE^9,15–17^. Moreover, it is shown that *T. molitor* can use PS and LDPE as a carbon source, breaking it down through the process of depolymerization to CO_2_ and H_2_O, due to bacteria present in the gut^11–14,18^. Gastrointestinal tract of *T. molitor* is characterized by a diverse microbiota members responsible for metabolism of different types of food^19^. The study by Yang et al.^11^ was the first to confirm the presence of PS degrading bacteria. The plastic biodegradation by *T. molitor* was mostly investigated for PS and the effect of this type of plastic on of the gut community members. The effect of PS on gut microbiota of *T. molitor* was monitored through a diet based exclusively on PS^11,14,20,21^ or mixed with bran^13,17^. Also, the ability of these insects to degrade LDPE has been demonstrated in several studies where the food source was only LDPE^9,16,22^, or co-fed with wheat bran^16,17^. Although larvae of *T. molitor* are capable of biodegrading PS, it has been shown that the PS only diet disrupts the life cycle of these insects^23^. Because polystyrene contain only hydrogen and carbon, it lacks nitrogen and micronutrients for optimal larval growth^23^. Futhermore, supplementation with bran, not only provides necessary nutrients, it also increase plastic consumption and biodegradation^12^ and reduce the adverse effect of PS^24^. Although studies on the ability of *T. molitor* to degrade PS and LDPE involve feeding these insects for a limited period of time, to our knowledge there are no data regarding the effect of long-term feeding with combination of bran and added different plastic materials on larval gut microbiota. Therefore, the aim of this study was to analyze the impact of three year co-fed plastic diet on the bacterial community of these self-sustaining laboratory populations of *T. molitor*, and to identify a potential plastic eating bacteria as well as mechanisms involved in the process of PS and LDPE biodegradation. The second goal was to compare and analyze the microbiota in all three self-sustainable cultures in the light of microbiological safety, and potential further use in sustainable circulatory systems, after biodegradation of plastics.

## Results

### Sequencing data processing

DNA from midguts of *T. molitor* larvae was sequenced, and provided from 125,000 to 172,000 original paired-end reads (*RawPE*). After quality filtering, 100,500 to 157,000 high-quality clean tags were obtained. These effective reads were assigned to 1 kingdom, 13 phyla, 23 classes, 74 orders, 55 families, 127 genera and 56 species in C group (Table 1), and it is obvious that the number in all ranks in PS group was similar to C group, while the lowest was in LDPE group. A high value of Good’s coverage index, ranged between 0.999 and 1.000, was achieved, which represents a good quality indicator of a real bacterial composition in all samples.

**Table 1:** Bacterial community in the **g**ut sample of *T. molitor* larvae feed with wheat bran (C), those feed with wheat bran and polystyrene (PS), and group feed with wheat bran and low-density polyethylene (LDPE).

Samples	Kingdoms	Phyla	Classes	Orders	Families	Genera	Species
C	1	13	23	74	55	127	56
PS	1	11	21	50	71	126	57
LDPE	1	10	19	41	63	93	42

### Relative abundance analysis at different OTUs level

**Fig. 1:**
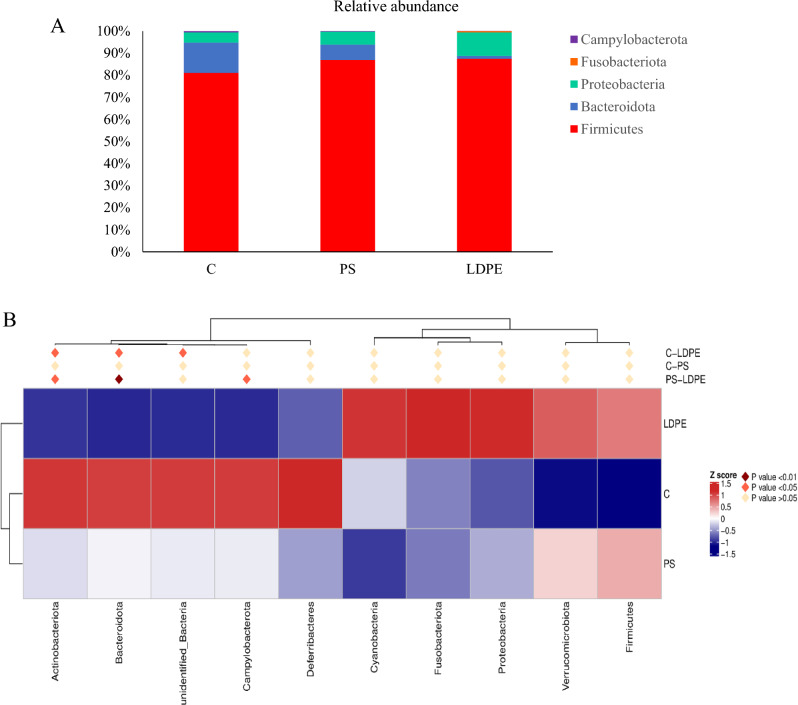
(**A**) Relative abundance (%) of 10 top phylum taxa found in each midgut sample of *T. molitor* larvae fed with wheat bran (C-control group), those fed with wheat bran and polystyrene (PS group), and group feed with wheat bran and low-density polyethylene (LDPE group); (**B**) Heatmap showing the differential abundance of bacterial phyla in all three groups. The color key relates the heatmap colors to the standard score (z-score), i.e., the deviation from the row mean in units of standard deviation above or below the mean. Asterisks indicate significantly different group abundances (p < 0.05).

Top 5 phyla detected in all analyzed groups were selected to form the distribution histogram presented in Fig. 1A**.** The most dominant was Firmicutes, followed by Bacteroidota and Proteobacteria, with total relative abundance over 90%, but differently distributed among groups. The lowest abundance of Bacteroidota phylum was noted in LDPE group with 1.3%, compared to 6.9% in PS, and 13.5% in the control group. Apparent changes in the distribution of the Proteobacteria were also observed between the groups, with 4.5% and 5.8% in C and PS respectively, while the highest abundance was noted in LDPE (10.6%).

To better visualize the differences between groups based on the phyla abundance heatmap was constructed and the obtained results were presented in Fig. 1B. On the basis of calculated z-scores, there were no significant differences in abundance of top 10 phyla between C and PS group. A significant decrease in the relative abundance of Bacteroidota and Actinobacteriota has been noted in LDPE group, compared to both C and PS group. Phylum Campylobacterota was differently abundant between PS and LDPE group, being significantly decreased in LDPE group.

Further, examination of the top 15 genera whose relative abundance (in either group) were ≥ 0.01, showed them to be differently distributed among groups (Table 2). Most of the changes were noted in mealworms co-fed with LDPE. Comparing to C group, consumption of LDPE have caused a significant increase of *Lactococcus.* Also, higher abundance of *Pediococcus* has been detected in LDPE group when compared to C and PS. However, relative abundance of *Lactobacillus*, although not reaching statistical significance (p = 0.051), was much lower in LDPE group when compared to C. No statistically significant changes in selected genera were noted between the C and PS group.

**Table 2:** Relative abundance of OTUs at the genus level (one-way ANOVA).

	C	PS	LDPE
*Spiroplasma*	0.210 ± 0.178	0.155 ± 0.144	0.080 ± 0.097
*Lactococcus*	0.061 ± 0.084	0.094 ± 0.078	0.239 ± 0.171*
*Clostridium *sensu stricto* 6*	0.034 ± 0.085	0.046 ± 0.108	0.003 ± 0.004
*Clostridium *sensu stricto* 1*	0.004 ± 0.009	0.002 ± 0.002	0.000 ± 0.000
*Enterococcus*	0.318 ± 0.295	0.498 ± 0.215	0.387 ± 0.134
*Pediococcus*	0.004 ± 0.004	0.004 ± 0.008^##^	0.100 ± 0.086**
*Weissella*	0.003 ± 0.006	0.005 ± 0.006	0.031 ± 0.065
*Lactobacillus*	0.053 ± 0.064	0.009 ± 0.006	0.002 ± 0.005*
*Dubosiella*	0.024 ± 0.036	0.004 ± 0.003	0.001 ± 0.002
*Bacillus*	0.006 ± 0.010	0.009 ± 0.021	0.019 ± 0.024
*Ileibacterium*	0.010 ± 0.023	0.000 ± 0.001	0.002 ± 0.005
*Limosilactobacillus*	0.014 ± 0.017	0.005 ± 0.004	0.001 ± 0.002
*Lachnospiraceae NK4A136 group*	0.012 ± 0.010	0.007 ± 0.015	0.000 ± 0.000
*Sebaldella*	0.000 ± 0.000	0.000 ± 0.000	0.005 ± 0.010
*Bacteroides*	0.006 ± 0.010	0.003 ± 0.002	0.001 ± 0.003

Results are presented as mean ± SD, statistically significant at *^#^ p < 0.05 **p < 0.01, ^###^***p < 0.005.

**Fig. 2:**
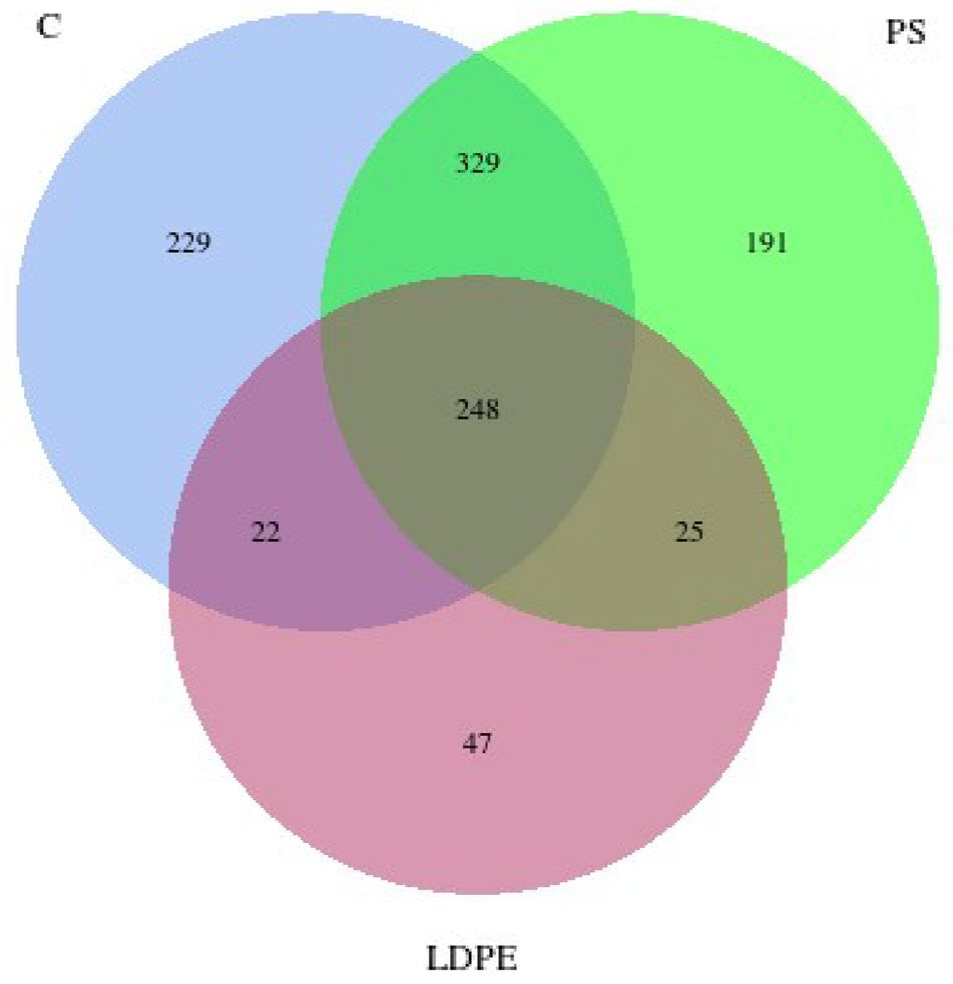
Venn diagram representing shared and common OTUs. All abbreviations are the same as in Fig. 1.

In order to visually represent the similarities and differences of the microbiota composition between C, PS, and LDPE, we counted the number of common and unique operational taxonomic units (OTUs) (Fig. 2). Venn diagram showed that all groups share stable core of OTUs most of which belong to class Bacilli, Clostridia and Bacteroidia, representing more than 75% of this core (Supplementary file). However, 229 OTUs were specific solely for the C (which makes 27.7% of all OTUs detected in this group) and 191 OTUs were specific for PS group (which makes 24.1% of all OTUs detected in this group). The lowest number of OTUs, only 47, was found in LDPE, representing 13.7% of all taxa detected in this group. While the control group shared 329 OTUs with PS, only 22 were common for the C and LDPE group.

### Gut community structure analysis of *T. molitor* larvae fed without and with PS and LDPE

**Fig. 3:**
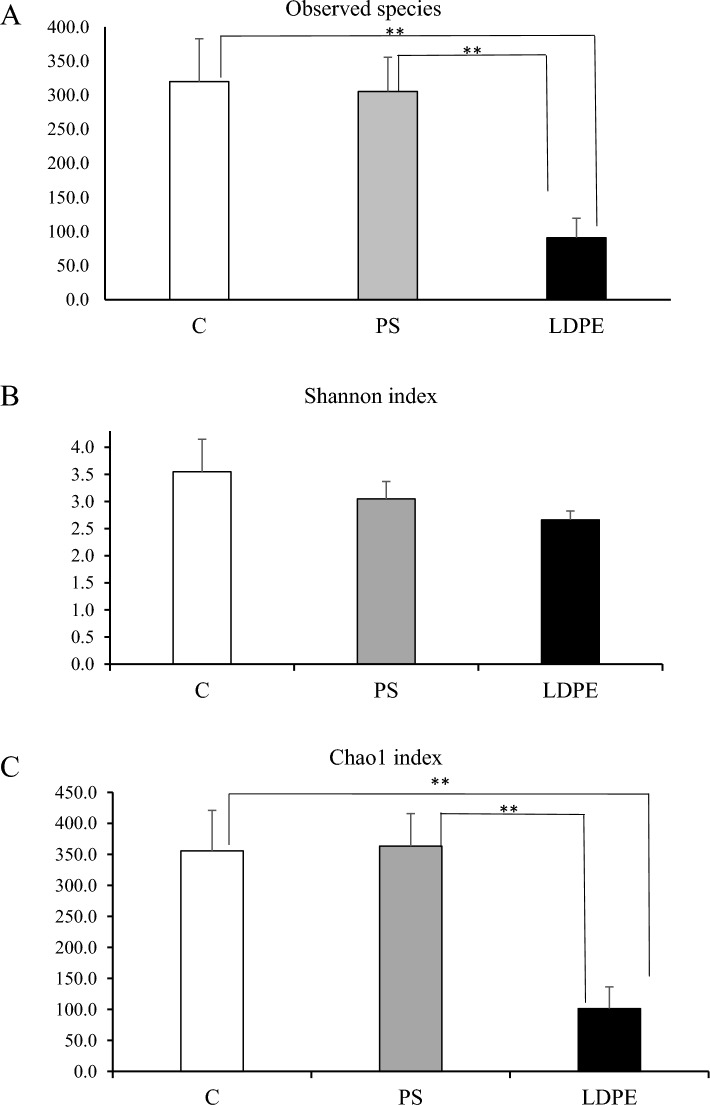
Alpha diversity indexes (**A**) Observed species, (**B**) Shannon index, (**C**) Chao1 index. Results are presented as mean ± SD, statistically significant at **p < 0.01. All abbreviations are the same as in Fig. 1.

To investigate the effects of polystyrene and low-density polyethylene on *T. molitor* gut microbiota richness (referring to number of taxonomic units) and evenness (distribution of abundances of the species), we performed the analysis of alpha diversity, presented as Observed species, Shannon and Chao1 indexes. Alpha diversity of amplicon sequencing data represents the first choice to assessing differences between examined environments^25^. In Fig. 3A the number of observed species is shown, and similar number of taxa were present in the C group and PS group while significant decrease was noted in LDPE, comparing to both C and PS. The Shannon index (Fig. 3B) indicating diversity of species (evenness), showed the highest value in the control group, while decrease was noted in PS and LDPE group of larvae, although these changes were not statistically significant. Based on the Chao1 index (Fig. 3C), it was apparent that consumption of LDPE caused a reduction of bacterial richness comparing to C group and PS group.

**Fig. 4:**
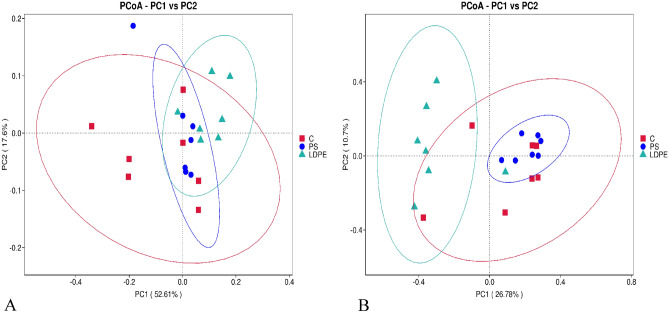
The 2D PCoA diagram based on the weighted (**A**) and unweighted (**B**) unifrac distance of bacterial communities. All abbreviations are the same as in Fig. 1.

Beta diversity was used as a comparative analysis of microbial community composition of analyzed samples from C, PS and LDPE group. Beta diversity metrics gives us insight into how samples differ from one another and reveal aspects of microbial ecology that could not be seen from analyzing of the composition of individual samples^26^. The differences between groups were examined through Principal coordinates analysis (PCoA) (Fig. 4A, B). In PCoA plot based on the Weighted UniFrac distance matrix it is obvious that there is less pronounced separation between the examined groups. However, PCoA plot based on the Unweighted UniFrac distance matrix (Fig. 4B), as being more sensitive to differences in low abundance feature, discovered more profound dissimilarities in microbial composition between LDPE group and both C and PS group.

To investigate the significance of inter-group community structure variability statistical tests Adonis and Anosim were performed. The Adonis test revealed significant differences for C-LDPE group (R^2^ = 0.237 (0.762), *p* = 0.005) and PS-LDPE group of larvae (R^2^ = 0.176 (0.823), *p* = 0.023) with variability of 23.7% and 17.6% respectively being responsible for the observed differences. The same was confirmed by the Anosim (R = 0.24, = 0.01 C-LDPE group and R = 0.19, *p* = 0.022 for PS-LDPE). There were no differences in community structure between C and PS group.

**Fig. 5:**
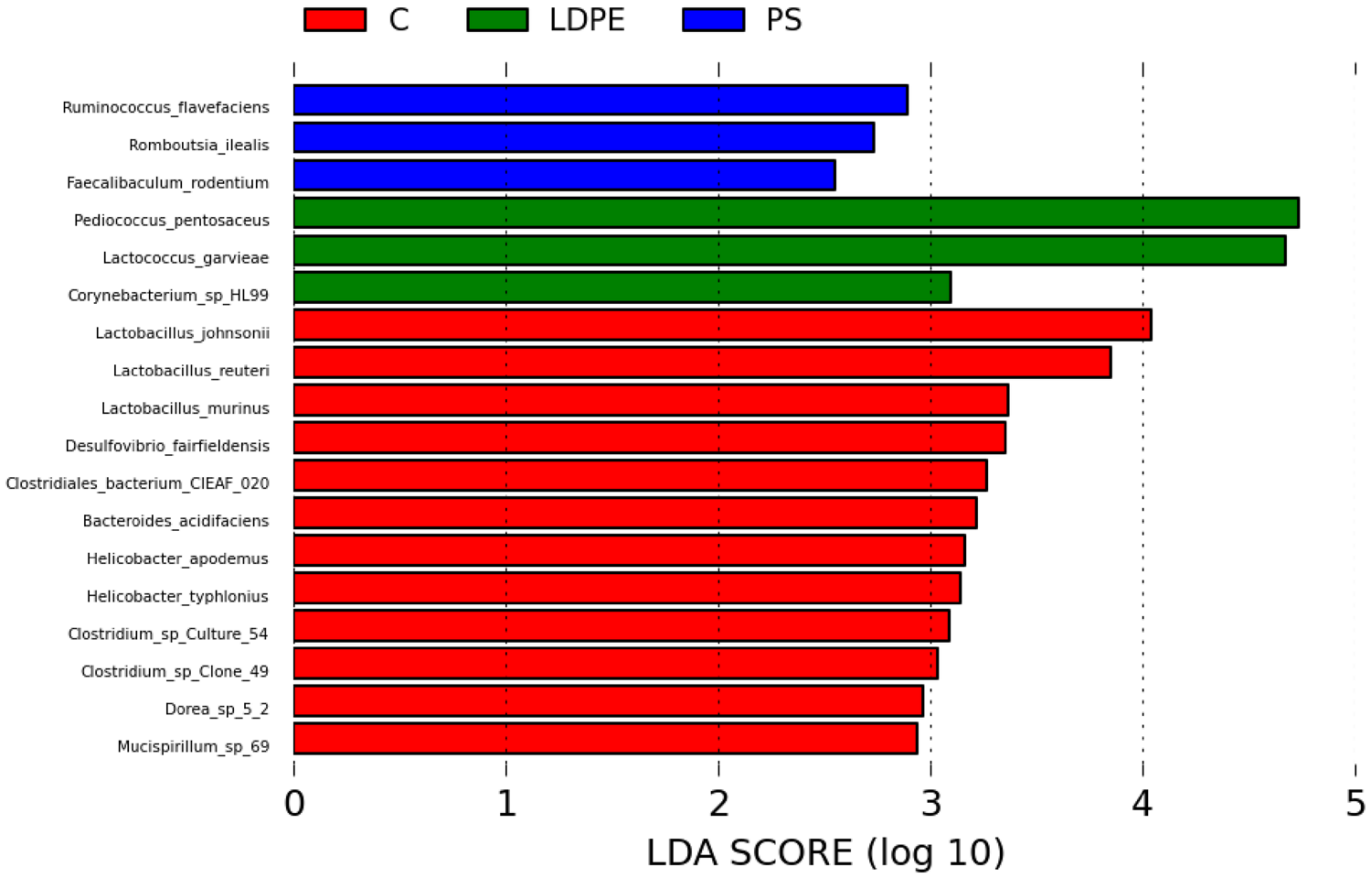
Linear discriminant analysis of effect size (LEfSe), based on OTUs at species level, and used to emphasize the differences between groups. All abbreviations are the same as in Fig. 1.

Linear discriminant analysis (LDA) of effect size (LEfSe) was used to identify biomarkers (OTUs here used at species level) to emphasize the differences between groups (Fig. 5). Total number of 18 species were found to be differently abundant. Most of them belong to the control group (12), while the same number of species was found in PS and LDPE group (3). Based on the LDA score ((log10) > 2.0) the most abundant species in the PS group were *Ruminococcius flavefaciens, Romboutsia ilealis* and *Faecalibaculum rodentium*, while strongly associated species for LDPE group were *Pediococcus pentosaceus, Lactococcus garvieae,* and *Corynebacterium* sp. HL 99. As a biomarker for the control group 12 species were observed, of which the most abundant belonged to *Lactobacillus* sp*.* and *Clostridium* sp*.*

**Fig. 6:**
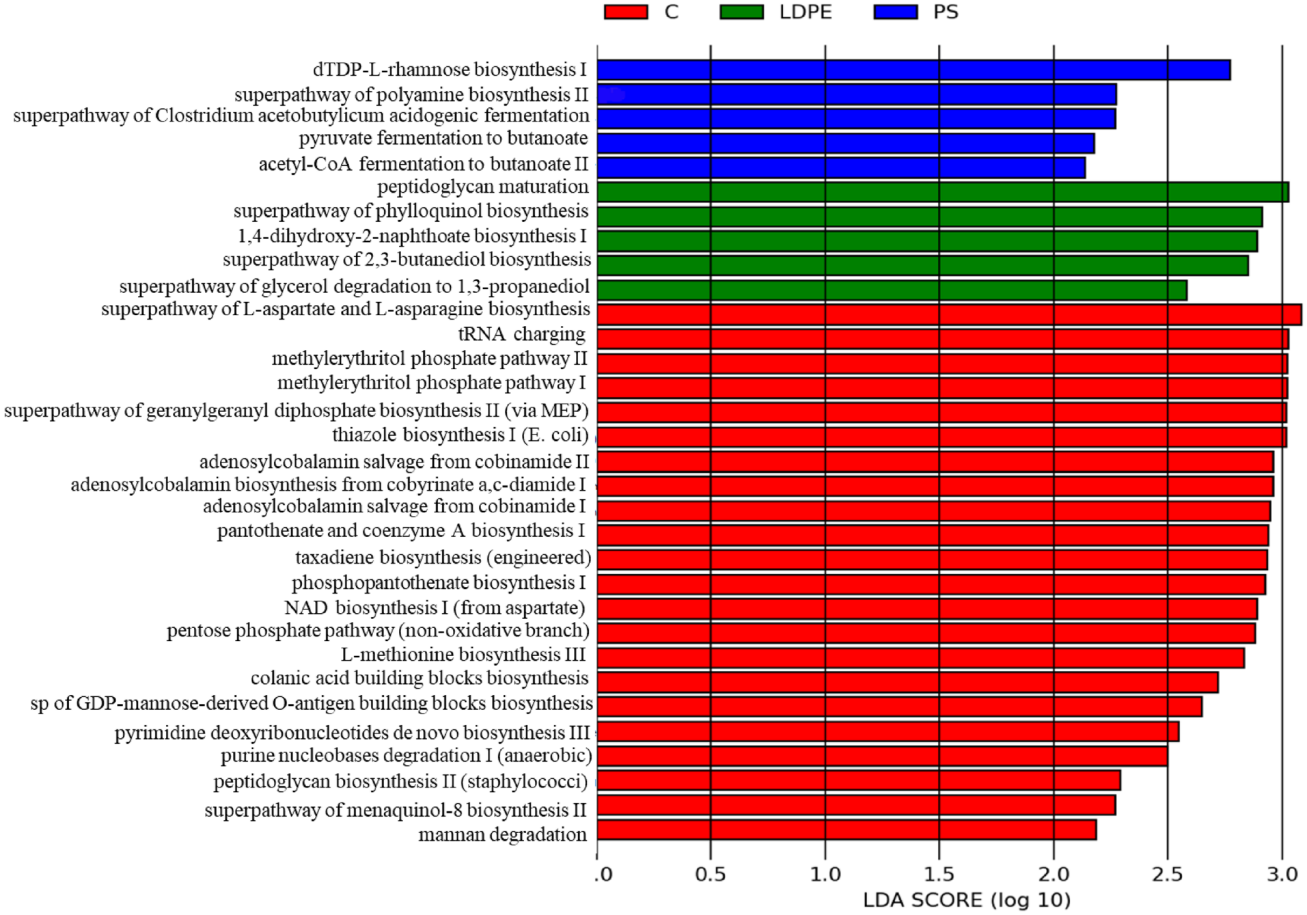
PICRUSt prediction of functional abundances, presenting only pathways or enzymes that reached an LDA significance threshold of > 2. All abbreviations are the same as in Fig. 1.

Prediction of functional abundances using PICRUSt, presenting only pathways or enzymes that reached an LDA significance threshold of > 2, (Fig. 6), for PS group showed five distinctive pathways: dTDP-L-rhamnose biosynthesis I, superpathway of polyamine biosynthesis II, superpathway of Clostridium acetobutylicum acidogenic fermentation, pyruvate fermentation to butanoate and acetyl-CoA fermentation to butanoate II. For LDPE group other distinctive pathways were linked: peptidoglycan maturation (meso-diaminopimelate containing), superpathway of phylloquinol biosynthesis, 1,4-dihydroxy-2-naphthoate biosynthesis I, superpathway of 2,3-butanediol biosynthesis, superpathway of glycerol degradation to 1,3-propanediol. In the control group, 22 pathways were predicted, different from PS or LDPE groups.

## Discussion

In this study, the effect of long term co-fed diet with PS and LDPE on gut microbiota of *T. molitor* was investigated. Examination of the relative abundance of OTUs at the phylum level indicated that most abundant phyla for all examined groups were Firmicutes, followed by Bacteroidota and Proteobacteria. Similar results were obtained when *T. molitor* larvae were fed with PS, corn straw and cabbage, where Firmicutes and Proteobacteria were the most abundant^21^. Also, Jiang et al.^27^ demonstrated that Firmicutes and Proteobacteria were the predominant phyla in the guts of 20 days PS-fed mealworms, and similar was shown in some other articles^14,22^. In addition to Firmicutes and Proteobacteria, other authors have noted that Tenericutes^28^ or Actinobacteria^29^ were also in high abundance in the gut of yellow mealworms. Although others studies reported low abundance of Bacteroidota in larvae fed with mixed bran (without plastic)^29,30^ our results showed that this phylum was the second most abundant in the control group. This was also noted in PS group, indicating that the abundance of the Bacteoidota was not much affected by the addition of polystyrene. However, ingestion of LDPE caused a significant decrease of Bacteroidota, Actinobacteriota and Campylobacterota, compared to control and PS group. It seems that the representatives of these phyla were much more impacted by the harmful effects of LDPE.

Based on the alpha diversity indices Observed species and Chao1 index it was obvious that different feeding diets have caused changes in gut microbiota of *T. molitor* larvae. In contrast to C and PS group, LDPE consumption caused a significant decrease in bacterial community richness. Venn diagram also showed that the control and PS group share more common OTUs, indicating that they are more similar to each other than to LDPE.

Also, relative abundance analysis at the genus level, showed that, *Spiroplasma*, *Lactococcus*, and *Enterococcus* were the most prevalent in all examined groups. The presence of these genera were also reported in gut of *T. molitor* fed with bran^14,17,28,30,31^, PS^17,21,32,33^, LDPE^9,15,32^ and with bran + PS/LDPE^14^. Although no differences were noted between the control and PS group on this OTU level, co-fed diet with LDPE caused a significant increase of *Lactococcus* and *Pediococcus* genera. Considering that *Lactococcus garvieae* and *Pediococcus pentosaceus* were differentially abundant in LDPE group (based on the LEfSe analysis) it can be assumed that these two species mainly contributed to the noted increase. Lecocq et al.^34^ identified *P. pentosaceus,* a lactic acid bacteria, with beneficial effect on *T. molitor* survival rate and development. This study also suggest that the relative abundance of this bacteria could promote the growth of other species, members of the order *Lactobacillales.* As *L. garviae* (member of the order *Lactobacillales*) was also abundant in LDPE group, this indicates the possible mutualistic relationship between these two species. Although, LDPE diet has led to a significant decrease in the bacterial richness in the gut of *T. molitor* larvae, we assume that *P. pentosaceus* besides having a protective effects on the *T. molitor* life cycle, might be, altogether with *L. garviae* involved in decomposition of LDPE.

Similarities between C and PS group of larvae were also confirmed by the analysis of beta diversity (PCoA) and statistical tests Anosim and Adonis. Since the structure of PS is similar to lignin and cellulose (biopolymers in bran) it seems that same group of bacteria are capable of degrading and using this type of polymers as a carbon source^35^. In light of that, it is worth considering *Ruminococcus flavefaciens*, as according to LEfSe, being differentially abundant in PS group, responsible for the degradation of PS, since the decomposition of lignocellulosic biomass by this bacteria has been confirmed in many studies^36,37^. Besides, feeding time with co fed PS diet is likely to have an effect on larval gut microbiota. Since PS group was very similar to control, this suggesting that gut bacteria could adapt well to the changing diet. On the other hand, gut microbiota composition of LDPE group was markedly different from either control or PS group. According to the LEfSe analysis in LDPE group were dominant species that belong to genera *Lactococcus, Pediococcus,* and *Corynebacterium*. Higher relative abundance of *Lactococcus* was also observed in the gut of larvae fed with PE + bran (28. 51%) compared to bran feeding group (15.34%)^17^. Also, *Corynebacterium* spp. plastic degrading capabilities were demonstrated in other studies^8,11^. However, higher prevalence of *L. garviae* was noted in worms fed on expanded polystyrene (EPS), and proposed that *L*. *garvieae* may be involved in EPS degradation^38^. Same group of authors noted that the abundance of *Pediococcus* was increased from 0.22% in group fed only with bran up to 18.25% in the PS + bran diet group. This is different from our results, in which this genus had a relative abundance of 0.4% in both control and PS group, but significantly higher abundance of 10% in LDPE group. The reason for the data discrepancies could be due to differences in plastic material being used (presence of additives and dyes) as well as rearing condition^13,39^. Also, individual variability in gut microbiota compositions could be the reason for different response. In this regard, great variability in bacterial community on phyla level was noted between two control groups of *T. molitor* purchased from the same source^28^. The relatively high variability of individual gut bacterial composition of *T. molitor* was also noted in another study^34^. Perhaps the biodegradation capacity of the intestinal bacterial community of these insects should not be observed universally, but the contribution of individual differences in gut microbiota should be considered^17^. It is apparent that the gut microbiota of *T. molitor* can be modulated by many factors. One of them is the environment, which is confirmed in the study of Cambon et al.^40^, where the authors showed that the bacterial community of laboratory reared population significantly differs from soil-reared insects. However, besides having a microbial community shaped by the environment and diet, an important factor in creating the diversity of symbiotic microorganisms in insect gut is the vertical transmission of bacteria from mother to offspring that could provide the essential adaptability of the host to new conditions^41,42^. For example, these microorganisms have a substantial role in host nutrient supply, physiological development, endurance and evolution^41^. In our three self-sustainable cultures, although PS and LDPE based diet did cause the emergence of the new bacterial species in comparison to control, all of them showed a certain degree of similarity in the composition of the gut microbiota. This could be explained by the presence of the heritable symbiotic bacteria, which constitute the stable core of the gut community, not being affected by the external factor yet important for fundamental physiological processes that are crucial for host survival.

While publicly available studies have already confirmed plastic degrading abilities of bacteria, also detected in our study, these results should be taken with caution, having in mind that genetic differences between different strains of the same bacterial species could contribute to individual variability and differences in biological properties^43–45^. Additional tests on the biodegrading properties of these bacteria must be conducted to confirm them.

Intending to examine which distinctive metabolomics pathways are connected to PS and LDPE group, we performed a predictive PICRUSt analysis. In PS group *Clostridium acetobutylicum* acidogenic fermenatation pathway was recognized. This bacteria is known to produce acetate and butanoate when grown at neutral pH on glucose is also known as an industrial producer of the organic solvents acetone, butanol, and ethanol^46^. Since polystyrene is soluble in the majority of organic solvents^47,48^, the breakdown of PS can be connected to the metabolic processes of acidogenic fermentation. In our metabolic prediction another two metabolic pathway were recognized in PS group, pyruvate fermentation to butanoate and acetyl-CoA fermentation to butanoate II. It is known that microbial decomposition of styrene, a monomer of PS, is decomposed by entering the TCA cycle^39,49^. Butanoate metabolism is indirectly linked the TCA cycle through pyruvate and acetyl-CoA^50^, pointing these two pathways to PS degradation. Additionally, butyric acid, a valuable volatile fatty acid (VFA) is the product of the butanoate (butyrate) metabolic pathway of the genus *Clostridium* (also differentially abundant in our PS group) through the process of the acidogenic fermentation^51^. VFAs are very important in various biotechnological processes such as the production of bioplastic and biodiesel, and for wastewater management^52^. However, since conventional chemical methods for the production of these VFAs are considered less economically and environmentally acceptable, sustainable biological sources are recognized as much better option^52^. Not only is acidogenic fermentation considered a good way to produce VFAs it has also been shown that the addition of plastic specially HDPE and PS increase total VFA yields by 28% and 47%, respectively^53^. Regarding that, it seems that *T. molitor* reared on bran + PS, represents a bioreservoir of valuable bacterial species that, besides the capacity to ingest and degrade PS, represent promising candidates for further application in biotechnology.

As for LDPE group, other five distinctive pathways were linked, like peptidoglycan maturation meso-diaminopimelate containing. This type of peptidoglycan was described in Corynebacterailes^54^ while *Corynebacterium* sp HL 99 (representative of order Corynebacteriales) was biomarker for the LDPE feed group in our case. Noticing a decrease in bacterial diversity in gastrointestinal tract in LDPE group, it can be assumed that in contrast to PS, LDPE type of plastic represent some kind of stressogenic factor. Therefore, it seems that peptidoglycan maturation is a reasonable mechanism of defense against different biotic and abiotic stressors^55^. Another metabolic pathway, the superpathway of 2,3-butanediol (BDO) biosynthesis, is done by many bacterial species, by fermentation of pyruvate via several intermediate compounds^56^. The role of this metabolic activity is in preventing toxic acetate accumulation^57^. The presence of this metabolite could also be from ethylene–vinyl acetate (EVA), present in our LDPE^58^. This copolymer is commercially used as an additive to increase strength and resistance to LDPE plastic^59^. We therefore hypothesize that this metabolic pathway could be one of the mechanisms for LDPE degradation. Also, 2,3 BDO is an important chemical with various applications in pharmaceuticals and industries, and the production of biofuels have attracted most of the attention. Considering that the small amounts of 2,3 BDO are being produced via petrochemical route and the tendency to reduce carbon dioxide emission, biological production of this chemical stands out as a promising alternative. Since *P. pentosaceus* was biomarker for LDPE group and was already recognized as a producer of the 2,3 BDO and 1,3-propanediol (1,3-PDO) after glycerol consumption^60^, it indicates that this bacteria might be a possible candidate for the 2,3 BDO production. Being aware that PICRUSt is only a predictive analysis, it is hard to be certain in the conclusions. This approach estimate the functional potential of the bacterial community while improving our understanding of the gut microbiota plasticity of *T. molitor* caused by the different feeding diet.

However, it is worth mentioning several bacterial species that showed their dominance in the C group of insects that were fed only with bran. According to the LEfSe, the most abundant species were *L. johnsonii, L, reuteri, L. murinus,* and *B. acidifaciens*, for which numerous studies have shown many probiotic properties^61–63^. Since *T. molitor* is considered as an alternative animal feed source, the presence of the beneficial bacteria emphasizes this group of larvae not only as a valuable source of protein and micronutrients, but as a source of possible probiotic strains.

Although further research needs to be done in order to determine the possible plastic degrading properties of bacteria detected in each group, and precise mechanism by which they utilize PS and LDPE, the obtained results indicate on how diverse application of this type of insect can be. Furthermore, it can be concluded that our cultures of T*. molitor* fed with added PS or LDPE might be useful in numerous biotechnological processes as a significant element in green technology, with a contribution to solving environmental pollution problems.

## Methods

### Insect rearing and sample preparation

The life cycle of *T. molitor* is short but can vary greatly due to environmental factors like temperature, humidity, food, and water. Larvae hatch from eggs, and become mature after 8 to 20 moltings, typically after 2–3 months. The mature larvae are of a light yellow brown color, 20 to 32 mm long, and weigh 130 to 160 mg. Mature larvae are used commercially as an alternative food source rich in proteins, lipids and polyunsaturated fatty acids for pets and livestock. Three self-sustaining laboratory populations of *T. molitor*, reared on wheat bran control (C group) and wheat bran with added polystyrene (PS group), and kitchen wrap as low-density polyethylene (LDPE group), have been reared in the Department of insect physiology and biochemistry for three years. Polystyrene and kitchen wrap cultures were originally fed with wheat bran. Healthy and vital larvae were randomly chosen and transferred to plastic containers with wheat bran and polystyrene, and wheat bran and kitchen wrap as new feeding substrate. Larvae were easily adapted to new feeding substrates, with approximately 4 generations per year. Recently, they were easily incorporated into the circular economy of zero waste insect farming in our institute, where containers with larvae and food substrate are stored in a rearing room with constant conditions: no light, 25 °C, 70% humidity, with optimal rearing density of 2 larvae/cm^2^, and the feeding rate was 1.5- 2 mg of feed/larvae/day. From all three self-sustaining *T. molitor* cultures, light yellow–brown mature larvae, 130 to 160 mg weight, were collected and sacrificed on ice. After scarification, guts were isolated and in each group 7 samples with seven guts in each sample, were collected and frozen on -80 °C for bacterial DNA extraction and sequencing.

### Bacterial DNA extraction and sequencing

Metagenomic DNA extraction from frozen gut samples was performed with ZR Fecal/Soil Microbe MiniPrep™ Kit (Zymo Research Corp., Irvine, CA USA), according to the manufacturer’s guidelines, and the concentration of isolated DNA was measured on Qubit™ fluorometer (Thermo Fisher/ Invitrogen, Waltham, MA USA). Samples were diluted to the concentration of 12 ng/μl, and 20 μl of final volume was sent to Novogene Company (Cambridge, United Kingdom). V3-V4 hypervariable regions of 16S rRNA gene were amplified using 341F (forward, 5ʹ–CCTAYGGGRBGCASCAG–3ʹ) and 806R (reverse, 5ʹ–GGACTACNNGGGTATCTAAT– 3ʹ) specific primers with the barcode. Sequencing libraries were generated, pooled and paired-end sequencing was performed on the Illumina NovaSeq 6000 platform, according to the required data amount. Paired-end reads were processed by cutting off the barcodes, adaptors, and primer sequences and merged using FLASH (V1.2.11)^64^. High-quality clean tags were obtained using Fastp (Version 0.23.1) software^65^. For species annotation at each taxonomic rank, the Silva Database^66^ was used in the Mothur algorithm^67^. A sufficient amount of sequencing data was achieved, based on the coverage index greater than 0.999 for each sample, reflecting the real composition of fecal microbiota^68^.

The data for this study have been deposited in the European Nucleotide Archive (ENA) at EMBL-EBI under accession number PRJEB78488 (https://www.ebi.ac.uk/ena/browser/view/PRJEB78488). Other data will be made available on request. The request should be addressed to Dr. Larisa Ilijin (lararid@ibiss.bg.ac.rs).

Before the alpha and beta diversity analysis, OTUs abundance data were normalized. The abundance information based on the top 10 taxa at phylum level, was used for the distribution histogram and done in Perl (5.26.2) through SVG function. Heatmap was drawn in R (Version 4.0.3) using the heatmap function. Venn diagram and Ternary plot were performed in R with Venn Diagram and vcd functions respectively. Alpha diversity (expressed through indices Observed species, Shannon, and Chao1 index), and beta diversity were calculated with QIIME (Version 1.9.1)^69^. Graphical representation of complex and multidimensional data was done with Principal coordinates analysis (PCoA) and displayed with R software^70^. In order to determine differences in community structure statistical tests which include Anosim, Adonis, were done with the vegan and ggplot2 package within R. All of the above-mentioned analysis were performed by Novogene Technology Co. Ltd.

Linear discriminant analysis effect size (LEfSe), and prediction of functional abundances PICRUSt2 were done using the Galaxy platform^71^.

### Data display and statistical analysis

Results are expressed as mean ± standard deviation (SD). For the statistical analysis the Statistica 7.0 (StatSoft Inc., Tulsa, OK) software was used. For the multiple comparisons between groups one-way ANOVA followed by Tukey’s test was used. A probability level less than 0.05 was considered significant.

## Supplementary Information

Supplementary Information.

## Data Availability

The data for this study have been deposited in the European Nucleotide Archive (ENA) at EMBL-EBI under accession number PRJEB78488 (https://www.ebi.ac.uk/ena/browser/view/PRJEB78488). Other data will be made available on request. The request should be addressed to Dr. Larisa Ilijin (lararid@ibiss.bg.ac.rs).
